# Network pharmacology-based identification of potential drug targets and bioactive compounds in *Lycii Fructus* (Gouqizi) for the therapeutics of Parkinson’s disease

**DOI:** 10.3389/fphar.2025.1714071

**Published:** 2026-01-12

**Authors:** Sufana Al Hashmi, Rana Adnan Tahir, Sheikh Arslan Sehgal

**Affiliations:** 1 Department of Biology, College of Science, Sultan Qaboos University, Muscat, Oman; 2 Department of Genomics and Bioinformatics, Cholistan University of Veterinary and Animal Sciences, Bahawalpur, Pakistan

**Keywords:** admet, bioinformatics, molecular docking, molecular dynamic simulations, network pharmacology, Parkinson’s disease, TCM

## Abstract

**Introduction:**

Parkinson’s disease (PD) is a common neurodegenerative disease characterized by the loss of dopaminergic neurons without any curable treatment. Various traditional Chinese medicines have been employed to manage the progression of PD. *Lycii Fructus* has demonstrated promising therapeutic potential in neurological disorders; however, the exact molecular mechanisms and specific bioactive compounds responsible for its effects remain unclear. The current study investigates the potential targets and binding potential of *Lycii Fructus* compounds against PD using bioinformatics approaches.

**Methods:**

Common disease and drug targets were retrieved and analyzed using protein-protein interactions and Cytoscape networks. Gene ontology enrichment analyses and KEGG pathway analyses were performed on the targets, followed by docking analyses to determine the binding potential of the compounds against these targets. Pharmacokinetic predictions and molecular dynamic simulations were performed to calculate the drug-likeness of compounds and extract the structural and residual fluctuations of top binding complexes, respectively.

**Results:**

The network pharmacology approach has identified AKT1, IL-1β, TNF, IL-6, and MAOB as key targets of PD. KEGG pathway analysis has shown that the ‘pathway of neurodegeneration-multiple diseases’ and ‘dopaminergic synapses’ are significant pathways of selected targets. Molecular docking studies have shown that the compounds cycloartenol, 24-methylenecycloartenol, lupeol acetate, 24-ethylcholesta-5,22-dienol, and 4α-methyl-24-ethylcholesta-7,24-dienol exhibited better binding potential against the scrutinized targets. AKT1 with 24-ethylcholesta-5,22-dienol (−11.5 kcal/mol), TNF with 4α-methyl-24-ethylcholesta-7,24-dienol (−10 kcal/mol), and MAOB with 24-ethylcholesta-5,22-dienol (−9.7 kcal/mol) exhibited a promising binding potential. The ADMET analysis of the selected five compounds reflects the potential of the drug candidate for effective PD therapies, as these compounds have a high drug-likeness score (0.76–0.78) and low drug-induced Neurotoxicity (<0.1).

**Conclusion:**

RMSD analysis of the top docked complexes showed that AKT1 - 24-ethylcholesta-5,22-dienol and MAOB-24-ethylcsecrholesta-5,22-dienol remained stable, whereas 4alpha-methyl-24-ethylcholesta-7,24-dienol exhibited fluctuations with TNF over 100 nanoseconds. These findings indicate that *Lycii Fructus* extracted compounds have high potential for targeting PD targets; further *in vitro* and *in vivo* experiments are necessary to assess their effectiveness in managing PD progression.

## 1 Introduction

Parkinson’s disease (PD) is a widespread neurodegenerative disease that is caused by the dysfunction of the dopaminergic nigrostriatal pathway, which in turn will reduce the motor function of the patient (Sehgal et al., 2018; Pajares et al., 2020). It is a progressive, prolonged neurodegenerative disease that mainly attacks the aging population; however, it might also affect young people (Beitz, 2014). The main reasons that lead to dopaminergic neuron death are still not clear. Studies show that almost 5%–10% of cases of PD are linked to genetic reasons, including a mutation in PARK genes that are responsible for coding the α-synuclein protein (Pajares et al., 2020). It has been reported that protein aggregation, autophagy-lysosome dysfunction, oxidative stress, mitochondrial dysfunction, and dopaminergic cell death can be associated with the development of PD (Li S. et al., 2020).

PD can be indicated by rigidity, bradykinesia, resting tremor, and postural imbalance (Cacabelos, 2017). The currently employed treatment for PD patients is levodopa (L-DOPA), designed to elevate dopamine levels in the brain. However, it has been noticed that using L-DOPA and other antiparkinsonian drugs might lead to the development of critical side effects such as symptomatic fluctuations and dyskinesia (Cacabelos, 2017; Li L. et al., 2020).

Network pharmacology (NP) is an area of study focused on system biology and multidirectional pharmacology (Shi et al., 2014). The first concept of network pharmacology was derived by Andrew L Hopkins in 2007 (Hopkins, 2007). NP employs various available computational resources to understand the molecular interactions of a specific drug in the cells (Chandran et al., 2017). NP is a holistic *in silico* method that reveals drug mechanisms by developing a protein-compound/disease-gene network. NP shifted the attention toward multi-target drugs instead of focusing on one target, one drug mode (Noor et al., 2022). NP has the potential to develop therapies for complex diseases influenced by several genes (Chandran et al., 2017).

For thousands of years, China has relied chiefly on traditional Chinese medicine (TCM) for treating different diseases. TCM has better effects in treating chronic and multifactorial diseases due to its synergistic effects on various targets, components, and channels when compared to single-drug treatments (Shi et al., 2014). Nowadays, TCM has been recognized as one of the primary components of the alternative or complementary medicine system after being under testing for several years (Zhang et al., 2013; Zhou et al., 2020). However, one of the main issues that casts doubt on the TCM system is the interaction between the targets. To overcome these complications and enhance the efficiency of TCM, it needs to be integrated with network pharmacology (Zhou et al., 2020).

Chinese people have used TCM to treat PD patients for more than 2000 years (Lin et al., 2021). Meta-analysis revealed that TCM adjuvant therapy enhanced PD patients' symptoms with fewer side effects (Zhang et al., 2015). TCM molecular mechanisms and their interactions with PD potential targets can be identified using a network pharmacology approach (Shen et al., 2022). *Lycii Fructus* (LF) (known as Gouqizi in China), also known as goji berry and wolfberry, is a mature dried fruit of *Lycium barbarum L.* LF was used as a therapeutic agent for many diseases due to its medicinal, preventative, and nutritional features. Many studies confirmed the antioxidative capability of LF polysaccharides (LFP), suggesting that LFP could be an efficient therapeutic or protective agent against PD (Zhang et al., 2020; Ali et al., 2025). Lin et al. highlighted LFP as a novel potential neuroprotective drug in PD models and explored its role in Iron homeostasis and lipid peroxidation-driven neurodegeneration. The study identified LF as an anti-PD potential drug for further clinical research (Lin et al., 2025). However, the exact mechanism by which LF controls PD progression remains unclear. The current study aims to elucidate the molecular mechanisms of LF against PD targets using network pharmacology and to identify an active formulation of LF, offering possible combinations for developing a potential therapy for PD patients. Additionally, this study will determine the binding potential and interactions of LF molecules with potential PD targets, as well as the pharmacokinetic properties of the selected compounds.

## 2 Materials and methods

**FIGURE 1:**
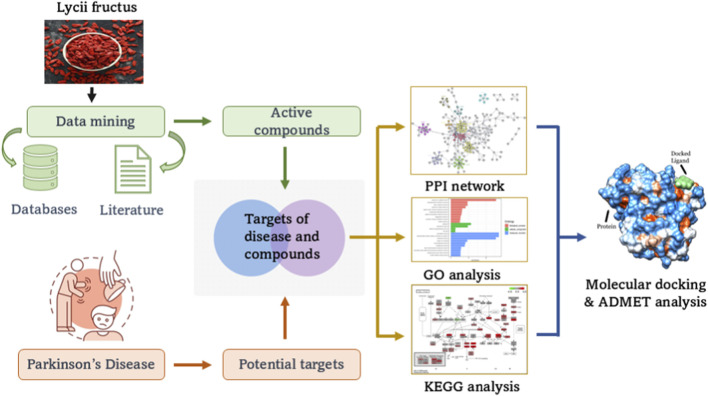
Illustration of the network pharmacology approach with *Lycii Fructus*.

The current study focuses on screening *Lycii Fructus* active compounds for PD therapeutics using a network pharmacology approach and molecular dynamics simulations, as shown in Figure 1.

### 2.1 Retrieval and screening of active compounds and PD-related targets

The phytochemicals of LF were obtained by using the Traditional Chinese Medicine System Pharmacology Database and Analysis Platform (TCMSP) (https://old.tcmsp-e.com/tcmsp.php) (TCMSP - Traditional Chinese Medicine Systems Pharmacology Database and Analysis Platform, 2025). The targets of PD were explored and identified from databases, including DisGENET (https://www.disgenet.org), GeneCards (https://www.genecards.org), OMIM (https://www.omim.org), and pharmGKB (https://www.pharmgkb.org), using the keywords “Parkinson’s disease”, “Parkinsonism”, and “Dopamine deficiency”. The common targets between the databases were identified by using the VENNY 2.1 (https://bioinfogp.cnb.csic.es/tools/venny).

### 2.2 Construction of drug component–target network

The LF-related targets were obtained from TSMSP, and gene names were converted to symbols using UniProt (https://www.uniprot.org). To visualize the intersection between the drug targets and PD targets, the VENNY 2.1 website was used. Cytoscape v3.10.2 (Shannon et al., 2003) software constructed an active compound–target network to visualize the interactions between active compounds and PD targets. The targets were identified as target nodes, while the LF ingredients served as source nodes. According to the degree value of nodes (i.e., the number of interactions), compounds were visualized in a circle layout, while targets were displayed in a grid layout, with the top targets localized in the center. Additionally, top targets and compounds were distinguished by different colors.

### 2.3 Construction of protein-protein interaction network

The intersection targets were used in the STRING v12.0 database (https://string-db.org/) to determine the protein interactions. The *Homo Sapiens* organism was used for the PPI network that will be further visualized by using several plug-ins: stringApp (v2.1.1) to integrate STRING results into Cytoscape, cytoHubba (v0.1) to analyze the network and determine its key targets, and yFiles Layout Algorithms (v1.1.4) to enhance the visual presentation of the network in Cytoscape v3.10.2 software. The top 5 hub targets were identified based on node degree, closeness, and literature evidence, and their corresponding PPI networks were constructed using the STRING database.

### 2.4 GO and KEGG enrichment analysis

The Gene Ontology (GO) enrichment analysis was carried out by g:Profiler (https://biit.cs.ut.ee/gprofiler), and the top 10 pathways were selected based on a threshold of 0.05 for Cellular Components (CC), Molecular Function (MF), and Biological Process (BP) values. Kyoto Encyclopedia of Genes and Genomes pathway enrichment analysis was performed using ShinyGO v0.81 (http://bioinformatics.sdstate.edu/go), and the related pathways to PD were identified and analyzed. The intersected targets of PD and LF compounds were used for GO and KEGG Enrichment analysis.

### 2.5 Molecular docking

The top key targets and active compounds of LF were selected for molecular docking. The 3D structure of target proteins was obtained from RCSB PDB (https://www.rcsb.org/), and any ligands or additional chains were removed by using UCSF Chimera 1.18 software (https://www.cgl.ucsf.edu/chimera/) (Uto et al., 2025). The 3D structures of active compounds were retrieved from PubChem (https://pubchem.ncbi.nlm.nih.gov/). Finally, the molecular docking was conducted by PyRx (Dallakyan and Olson, 2015) with nine output models and an exhaustiveness of 10. The binding energy between key targets and active compounds was used to assess the binding potential of compounds, followed by binding interactions with target proteins using UCSF ChimeraX (Meng et al., 2023). LigPlot^+^ (Laskowski and Swindells, 2011) was used to visualize the interacting residues of the docked complexes.

### 2.6 Molecular dynamic simulations

Molecular Dynamics (MD) simulations were performed to investigate the structural fluctuations and stability of the docked complexes for 100 ns using the Desmond tool from the Schrodinger LLC suite (Bowers et al., 2006). Compared to molecular docking, which provides a static view of ligand binding, MD simulations employ Newtonian equations of motion to assess the binding behavior under physiological conditions (Firdoos et al., 2023). Prior to the simulations, the protein–ligand complexes were minimized and optimized using the Maestro Preparation Wizard, and the systems were prepared using the System Builder tool. The Transferable Intermolecular Interaction Potential 3 Points (TIP3P) water model with an orthorhombic box was employed. Counterions were added to neutralize the system, and the OPLS 2005 force field was applied (Shivakumar et al., 2010). Sodium chloride (NaCl) was added to mimic the physiological conditions. System energy minimization was performed in five stages, each consisting of 3000 steepest descent cycles and 5000 conjugate gradient cycles. In the first stage, hydrogen atoms in water were minimized; in the second stage, water and ions were minimized; in the final stage, all solvent and solute molecules were minimized. After model relaxation, MD simulations were conducted in the NPT ensemble at 303.3 K and 1 atm. Structural fluctuations and stabilities were monitored throughout the simulation, and trajectory data were extracted every 200 ps. Root mean square deviation (RMSD), Root mean square fluctuations (RMSF), and protein-ligand contact analysis were conducted to extract the structural fluctuations. MD trajectory analysis was also performed, with principal component analysis (PCA) used to explore protein conformational changes upon compound binding. The eigenvalues of the receptor, specifically, PC1, PC2, and PC3, were plotted against the corresponding eigenvector indices for the first 20 motion modes. The residue cross-correlation matrix was also plotted to reveal the correlation between each residue and all the other throughout the simulation period.

### 2.7 ADMET analysis

ADMET Lab 3.0 (https://admetlab3.scbdd.com/) and TSMSP databases were used to assess the pharmacological and ADMET (Absorption, Distribution, Metabolism, Excretion, and Toxicity) properties of LF compounds. Initial compound screening was conducted based on molecular weight (MW), LogP, and drug likeness (DL) of all LF compounds, with the final selected compounds subsequently reassessed following docking analysis.

## 3 Results

### 3.1 Screening of active compounds and PD-related targets

**FIGURE 2:**
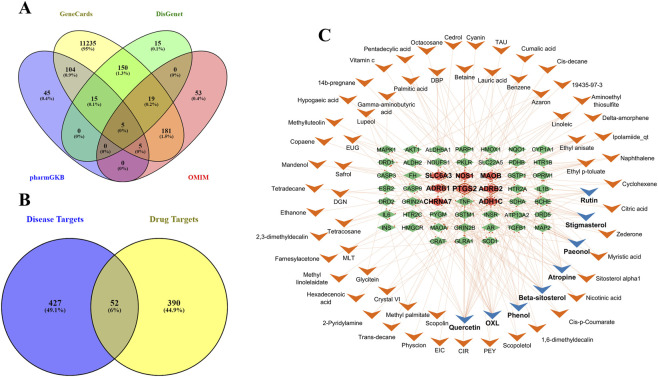
Venn diagrams illustrating shared and disease-specific targets. **(A)** The intersection between PD-related targets from biological databases, i.e., GeneCards, DisGenet, pharmGKB, and OMIM. **(B)** Intersection between disease and drug targets, revealing a total of 52 shared targets associated with PD. **(C)** The active compounds in *Lycii Fructus* that interact with PD targets, visualized in a Cytoscape network. Top targets are marked with bold labels and distinguished by different colors; compounds with more interactions are shown with a blue arrowhead shape, while targets with high interactions are highlighted with a light red diamond shape.

The objective involves the extraction and screening of active compounds and PD-related targets to uncover the compound-target interactions in the network pharmacology framework. All LF constituents were examined in the literature and databases, including TCMSP and TM-MC. However, a total of 188 comprehensive chemical and ADMET properties of LF components were obtained from TSMSP. Four databases were used to ensure thorough identification of PD-related targets: 11,714 genes were obtained from GeneCards, 204 from DisGenet, 174 from PharmGKB, and 66 from OMIM. The 479 common genes were selected from the intersection in the Venn diagram, as shown in Figure 2A.

### 3.2 Construction of drug component–target network

The overlapping molecular targets of the drug and disease identified potential therapeutic nodes, and the multi-target, multi-compound interactions are visualized in a Cytoscape network. The overlap between drug targets and PD targets is visualized in a Venn diagram as presented in Figure 2B. A total of 52 genes were identified that interact with 66 active compounds. The active compounds-targets network indicates that quercetin (degree = 19), OXL (degree = 15), phenol (degree = 10), beta-sitosterol (degree = 10), and atropine (degree = 10) are the compounds with the most significant number of interactions with targets. Targets such as PTGS2, ADRB2, SLC6A3, ADHIC, and MAOB exhibited the most interactions with LF components. The network shown in Figure 2C includes 119 nodes, with a blue arrowhead indicating the top active compounds and a light red diamond shape highlighting the primary targets among them. A total of 224 edges depict interactions among various nodes, and some long-named compounds were abbreviated for more precise network visualization.

### 3.3 Construction of protein-protein interaction network

**FIGURE 3:**
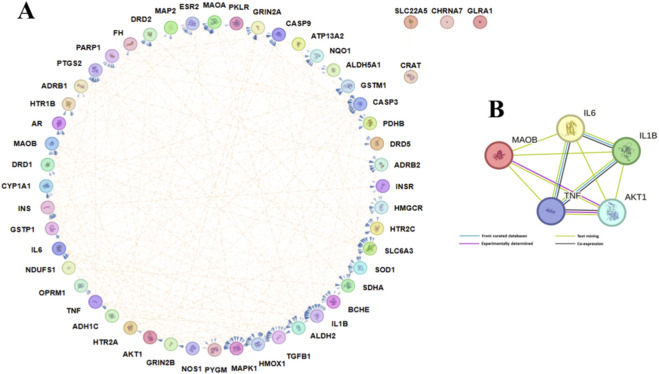
Protein–Protein Interaction Networks. **(A)** PPI network highlighting the potential 52 targets generated by STRING and customized using the Cytoscape tool; edges represent the interactions between proteins. **(B)** PPI of the top 5 PD targets according to their degree generated by STRING; different edges represent different evidence types.

A PPI network was constructed from the STRING database using an interaction score threshold of 0.400 (medium confidence) to identify interacting proteins. The PPI network built by Cytoscape shows a total of 52 nodes, 322 edges, an average node degree of 10.4, an average local clustering coefficient of 0.592, 111 expected edges, and a PPI enrichment p-value of less than 1.0e-16 (Figure 3A). The top 10 targets were identified based on degree values, and five (5) of them were selected based on literature evidence associated with PD. The STRING scores of the top targets, AKT1, IL6, TNF, and IL1B, ranged from 0.864 to 0.998, whereas MAOB showed a lower score (0.418–0.454) but demonstrated strong literature-based evidence. Interactions of the top 5 targets were visualized by the STRING database, as shown in Figure 3B. AKT1 (serine/threonine-protein kinase) has the highest degree value, followed by IL1B (Interleukin-1 beta), TNF (Tumor necrosis factor), IL6 (Interleukin-6), and MAOB (Amine oxidase [flavin-containing] B).

### 3.4 GO and KEGG enrichment analysis

**FIGURE 4:**
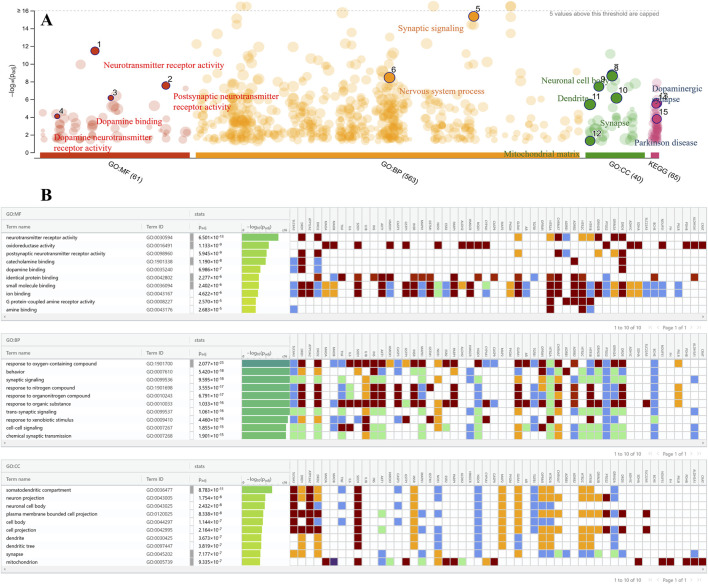
Gene ontology enrichment analysis. **(A)** Significant GO terms and KEGG pathways are depicted and labelled in the bubble chart. **(B)** Top ten MF, BP, and CC terms are shown along with their target genes sorted on -log_10_(P_adj_). The color of the bars represents the significance level based on -log_10_(P_adj_), and the heatmap displays the corresponding genes for each term.

A set of 52 intersection genes was used to conduct GO analysis using g: Profiler to highlight the role of these targets in pathways, molecular functions (MF), biological processes (BP), and cellular components (CC) (Figure 4A). A total of 49 MF, 571 BP, and 38 CC pathways were enriched at a significant level of p ≤ 0.05.

MF analysis revealed that the most significantly enriched terms among the targeted genes are neurotransmitter receptor activity, oxidoreductase activity, dopamine binding, and postsynaptic neurotransmitter receptor activity, all of which are closely related to MF implicated in PD. The significant BP terms include response to oxygen-containing compounds, behavior, synaptic signaling, trans-synaptic signaling, and chemical synaptic transmission, with high-log values of 2.077 × 10-23, 5.420 × 10^−18^, 9.595 × 10^−18^, 1.061 × 10^−16^, and 1.901 × 10^−15,^ respectively.

The somatodendritic compartment, neuronal projection, neuronal cell body, synapse, and mitochondria were the key CC terms among target genes that play a substantial role in PD development. The pathway analysis also shows that the dopaminergic synapses and PD are the top pathways for these targeted genes. The top 10 GO enrichment terms of MF, BP, and CC analyses are presented in Figure 4B.

**FIGURE 5:**
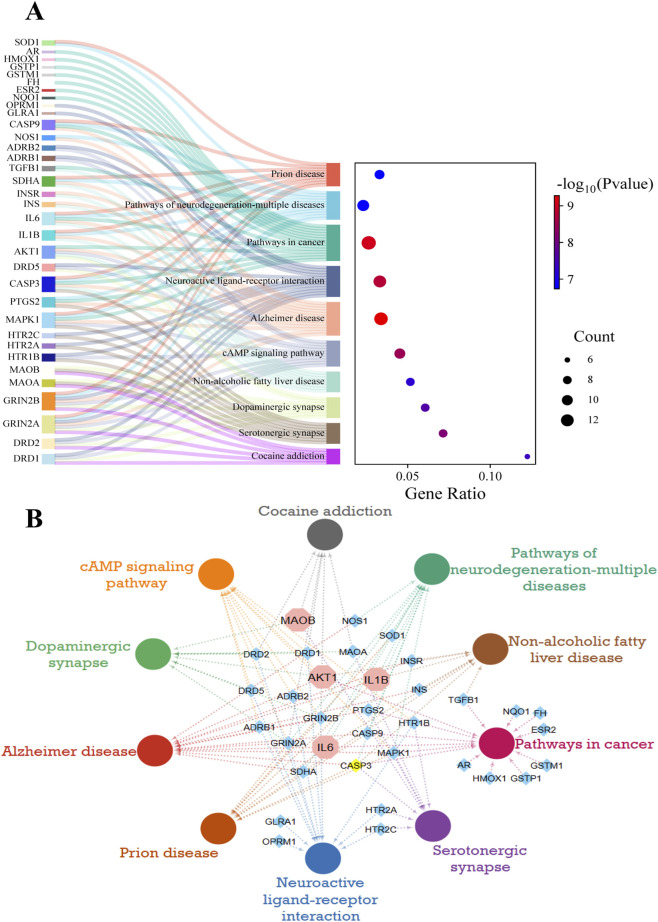
KEGG pathways analysis of potential targets. **(A)** Sankey and dot diagrams present the involvement of target genes in the top 10 pathways along with the significance level. The dot size represents the number of associated genes, while the color shifts from blue to red as the -log10 (p-value) increases. **(B)** Interactions of the top 10 KEGG pathways with related PD targets. Pathways are shown as circles in different colors. Blue diamonds indicate genes, while the top selected targets are in octagons.

KEGG Enrichment analysis identified 210 pathways for these target genes, and the top 10 are further analyzed. The top 10 pathways, including Cocaine addiction, serotonergic synapse, non-alcoholic fatty liver disease, dopaminergic synapse, CAMP signaling pathway, Alzheimer’s disease (AD), prion disease, neuroactive ligand-receptor interaction, pathways of neurodegeneration-multiple diseases, and pathways in cancer based on -log_10_ (p-value) and number of genes, are chosen and represented in Figure 5A. Pathway analysis highlights significant pathways, including AD, with a high number of genes and a high -log_10_ (p-value) 9, and dopaminergic synapses, with a high enrichment score. The interactions of targeted genes within these pathways were also mapped out and shown in Figure 5B. PD targets extracted from the PPI network are also analyzed, and their roles in these top-enriched pathways are determined. It has been observed that the MAOB gene is involved in dopaminergic synapses, serotonergic synapses, and cocaine addiction pathways. AD pathways involve (IL6, AKT1 and IL1B); prion disease involves (IL6 and IL1B); and pathways of neurodegeneration-multiple diseases involve (IL6, AKT1 and IL1B). Other pathways include pathways in cancer (IL6 and AKT1), non-alcoholic fatty liver disease (AKT1 and IL1B), and the CAMP signaling pathway (AKT1). The significant enrichment values for the AD pathway (1.5 × 10^−12^), the dopaminergic synapses (2.2 × 10^−10^), and the neurodegeneration-multiple diseases pathways (4.3 × 10^−9^) indicate the key roles of target genes in neurodegenerative disorders, particularly PD.

The KEGG graphs of dopaminergic synapses and neurodegeneration-multiple diseases pathways were plotted and analyzed to identify the role of key genes in these pathways (S1 Figure 1) and (S1 Figure 2).

### 3.5 Molecular docking

**FIGURE 6:**
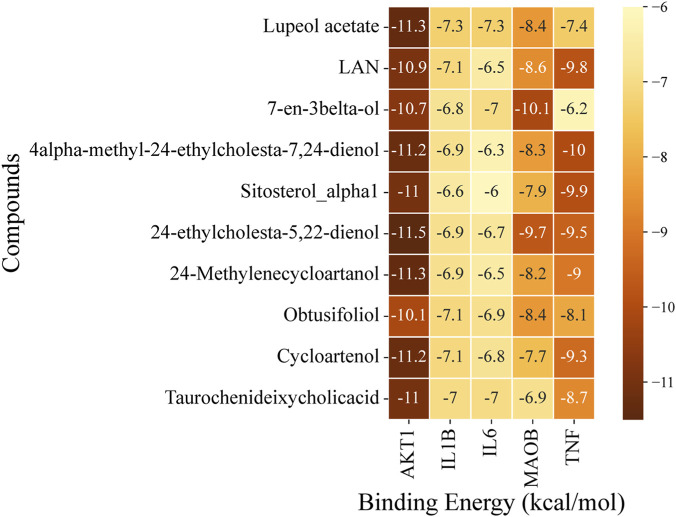
Heatmap represents the docking scores of the top 10 compounds against selected potential PD targets.

Molecular docking analysis of LF active compounds with selected targets, i.e., AKT1, IL1B, IL6, MAOB, and TNF, was conducted to determine the binding potential of compounds with targets. The 3D structures of proteins were retrieved from RCSP PDB as follows: AKT1 with PDB ID of 3O96 (446 aa), IL1B with PDB ID of 1IOB (153 aa), IL6 with PDB ID of 8QY5 (212 aa), MAOB with PDB ID of 1S2Q (520 aa), and TNF with PDB ID of 1TNF (157 aa). The receptor proteins and compounds were prepared, followed by the docking analysis using PyRx. Docking analysis of all 166 LF-extracted compounds was performed against the selected potential targets. The top five compounds, including Cycloartenol, 24-methylenecycloartanol, 24-ethylcholesta-5,22-dienol, 4alpha-methyl-24-ethylcholesta-7,24-dienol, and Lupeol acetate, with the least binding energy values, are shortlisted, and their 2D chemical structures are presented in (S1 Figure 3). The binding interactions of docked complexes were visualized and analyzed by UCSF ChimeraX 1.9. The binding scores of the top compounds for each target are presented in a heatmap (Figure 6).

Docking analysis exhibited that AKT1 has high binding potential with all the top compounds. 24-ethylcholesta-5,22-dienol exhibited the least binding energy with AKT1 (−11.5 kcal/mol), and with other target proteins, MAOB (−9.7 kcal/mol), TNF (−9.5 kcal/mol), IL1B (−6.9 kcal/mol), and IL6 (−6.7 kcal/mol). The binding energies of 24-methylenecycloartanol with top targets are: AKT1 (−11.3 kcal/mol), TNF (−9 kcal/mol), MAOB (−8.2 kcal/mol), IL1B (−6.9 kcal/mol), and IL6 (−6.5 kcal/mol). Lupeol acetate exhibited the least binding energy with AKT1 (−11.3 kcal/mol), followed by MAOB (−8.4 kcal/mol), TNF (−7.4 kcal/mol), and IL1B and IL6, which had the same binding energy (−7.3 kcal/mol). Cycloartenol has the lowest binding energies with AKT1 (−11.2 kcal/mol), TNF (−9.3 kcal/mol), MAOB (−7.7 kcal/mol), IL1B (−7.1 kcal/mol), and IL6 (−6.8 kcal/mol). The least binding energy of 4alpha-methyl-24-ethylcholesta-7,24-dienol after AKT1 (−11.2 kcal/mol), is TNF (−10 kcal/mol) followed by MAOB (−8.3 kcal/mol), IL1B (−6.9 kcal/mol), and IL6 (−6.3 kcal/mol). Compound 7-en-3beta-ol exhibits a higher binding energy (−10.1 kcal/mol) with MAOB than the selected compounds; however, the top compounds were selected based on their overall scores across all proteins.

**FIGURE 7:**
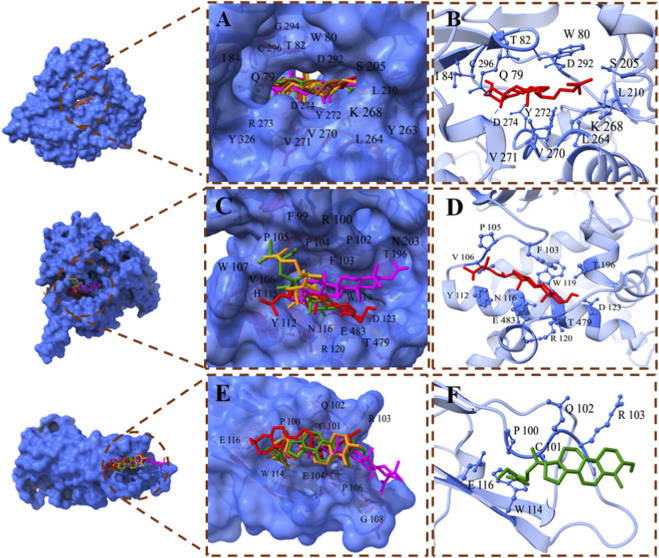
Binding interactions of the top compounds. **(A)** Binding interactions of AKT1: all compounds bind within the same binding groove of AKT1. **(B)** The binding interacting residues of AKT1 with 24-ethylcholesta-5,22-dienol, which has the least binding energy among other complexes. **(C)** Binding interactions of MAOB: four compounds fit within the same binding pocket, while 24-Methylenecycloartano binds to MAOB in another site. **(D)** Interacting residues of the MAOB - 24-ethylcholesta-5,22-dienol complex exhibit high binding affinity. **(E)** Binding interactions of TNF: All compounds bind to TNF at the same binding site. **(F)** Binding interactions of 4alpha-methyl-24-ethylcholesta-7,24-dienol with TNF; the complex with the least binding energy. Compounds are displayed in a stick style with different colors.

The binding interactions of top docked complexes were determined and analyzed based on the least binding energy values of compounds with each target. The top three targets, including AKT1, TNF, and MAOB, have high binding potential with top compounds, such as 24-ethylcholesta-5,22-dienol, 4α-methyl-24-ethylcholesta-7,24-dienol, and 24-ethylcholesta-5,22-dienol, respectively, and were chosen for interaction analysis (Figures 7A–E).

As mentioned previously, AKT1 has a high binding affinity with all top compounds, and all compounds fit within the same binding pocket, as shown in Figure 7A. The AKT1 complex with 24-ethylcholesta-5,22-dienol complex demonstrates the interacting residues Gln79, Trp80, Thr82, Ile84, Ser205, Leu210, Leu264, Lys268, Val270, Val271, Tyr272, Asp274, Asp292, and Cys296 (Figure 7B). The binding analysis reveals that the ligand is deeply buried within the protein binding pocket, and the residues Trp80, Ile84, Leu210, Leu264, Val170, and Val171 have hydrophobic interactions with the ligand, while other residues form hydrophilic interactions. Asp274 and Asp292 participate in basic interactions with 24-ethylcholesta-5,22-dienol. Additionally, Lys268 and Arg273 interact with 24-ethylcholesta-5,22-dienol via acidic interactions (S1 Figure 4A). Cys296 forms a hydrogen bond with the hydroxyl group and acts as a backbone donor (S1 Figure 4B).

MAOB also has promising binding potential; however, only four compounds bound in the same pocket, while 24-Methylenecycloartanol is close to them (Figure 7C). The 24-ethylcholesta-5,22-dienol has high binding potential with MAOB, and its interacting residues are Phe103, Pro105, Val106, Tyr112, Asn116, Trp119, Arg120, Asp123, Thr196, Thr479, and Glu483 (Figure 7D). The binding interactions analysis revealed that the aromatic part of the ligand binds inside the protein pocket and forms several hydrophilic interactions with polar (Tyr112, Asn116, Thr195, Thr196, Thr478, and Thr479), basic (Arg120), and acidic (Asp123 and Glu483) residues (S1 Figure 5A). Analysis suggests a stable and specific polar environment that accommodates the aromatic ring, where Asp123 and Thr196 form a hydrogen bond with the hydroxyl group, and Thr196 acts as a sidechain acceptor (S1 Figure 5B). On the other hand, the chain region of the ligand binds at the surface of MAOB, interacting with hydrophobic residues including Phe103, Pro104, Pro105, Val106, Trp107, and Trp119.

TNF also shows good binding affinity with top compounds, and all of them bind on the same site (Figure 7E). The complex TNF-4alpha-methyl-24-ethylcholesta-7,24-dienol is visualized in Figure 7F, and the interacting residues are Pro100, Cys101, Gln102, Arg103, Trp114, and Glu116. Binding analysis shows that 4alpha-methyl-24-ethylcholesta-7,24-dienol binds at the surface of the TNF protein. The interactions include several hydrophilic interactions: polar (Cys101 and Gln102), acidic (Glu104 and Glu116), and basic (Arg103). The residues Pro100 and Trp114 interact hydrophobically with the ligand molecule (S1 Figure 6).

### 3.6 Molecular dynamic simulations

**FIGURE 8:**
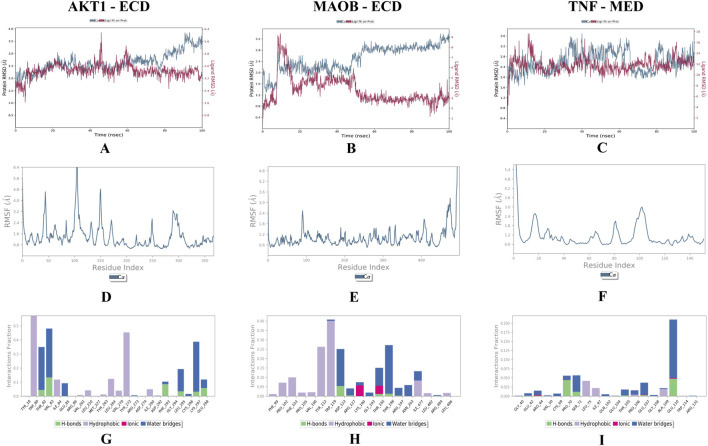
MD simulations results; **(A–C)** shows the RMSD plots of docked complexes, **(D–F)** shows the RMSF graphs present the residual fluctuations of the docked complexes, and **(G–I)** represent the Protein-Ligand Contacts throughout the simulation. **(A)** AKT1 - 24-ethylcholesta-5,22-dienol. **(B)** MAOB - 24-ethylcholesta-5,22-dienol. **(C)** TNF-4alpha-methyl-24-ethylcholesta-7,24-dienol. The X-axis shows time in nanoseconds (ns), the left Y-axis shows protein RMSD, and the right Y-axis shows ligand RMSD. **(D)** AKT1 - 24-ethylcholesta-5,22-dienol. **(E)** MAOB - 24-ethylcholesta-5,22-dienol. **(F)** TNF-4alpha-methyl-24-ethylcholesta-7,24-dienol. The X-axis shows the residues, while RMSF (Å) is presented on the y-axis. **(G)** AKT1 - 24-ethylcholesta-5,22-dienol. **(H)** MAOB - 24-ethylcholesta-5,22-dienol. **(I)** TNF-4alpha-methyl-24-ethylcholesta-7,24-dienol.

MD simulations of the top docked complexes (AKT1 - 24-ethylcholesta-5,22-dienol, TNF - 4alpha-methyl-24-ethylcholesta-7,24-dienol, and MAOB - 24-ethylcholesta-5,22-dienol) were performed to identify structural fluctuations and stability over 100 ns (Figures 8A–I).

The RMSD values for the AKT1 - 24-ethylcholesta-5,22-dienol complex indicate that the protein remained stable throughout the simulation, with an average RMSD of 2.5 Å and only minor fluctuations over 15 ns. The ligand also remained stable, with RMSD values ranging from 2.7 Å to 3.3 Å (Figure 8A). These results suggest that the AKT1-24-ethylcholesta-5,22-dienol complex is stable under physiological conditions. For the MAOB-24-ethylcholesta-5,22-dienol complex, the protein RMSD ranged from 1.5 Å to 3.1 Å and stabilized after 18 ns. The ligand RMSD began at 0.5 Å and peaked at 3.1 Å, with minor fluctuations observed between 05 ns and 15 ns, followed by stabilization (Figure 8B). Overall, the protein-ligand complex remained stable with only slight fluctuations during the simulation. However, the RMSD plot of the TNF-4alpha-methyl-24-ethylcholesta-7,24-dienol complex shows several fluctuations, with RMSD values ranging from 1.5 Å to 2.5 Å for the protein (Figure 8C).

In the AKT1 - 24-ethylcholesta-5,22-dienol complex, five prominent peaks appeared in the RMSF plot, with residue fluctuations xceeding 3.2 Å in the regions 1–10, 40–50, 95–110, 140–157, and 285–305 (Figure 8D). The RMSF plot of the MAOB-24-ethylcholesta-5,22-dienol complex shows only a single main peak at the terminal end, along with a minor peak at positions 90–95 with an RMSF value of 2.4 Å (Figure 8E). The TNF-4alpha-methyl-24-ethylcholesta-7,24-dienol complex exhibited significant fluctuation exceeding 3.0 Å at the N-terminus (residues 0–5), while other minor peaks of residual fluctuations at positions 14–20 and 95–108 were below 3.0 Å (Figure 8F).

The protein-ligand contact analysis of the AKT1-24-ethylcholesta-5,22-dienol complex indicated that specific residues maintained significant interactions with the ligand, suggesting enhanced complex stability. Specifically, residues Thr-82, Val-83, Phe-293, Leu-295, Lys-297, and Gln-298 formed hydrogen bonds with the ligand and interacted consistently throughout the simulation (S2 Figure 1), highlighting their key role in binding (Figure 8G). In the MAOB-24-ethylcholesta-5,22-dienol complex, the ligand formed hydrogen bonds with the residues Asp-123, Arg-127, Thr-195, Thr-196, and Arg-197 (Figure 8H). Tyr-112 and Trp-119 maintained continuous hydrophobic interactions with the ligand (S2 Figure 2). In the TNF-4alpha-methyl-24-ethylcholesta-7,24-dienol complex, most interactions were present for less than 30% of simulation duration. However, the ligand formed hydrogen bonds with several residues, including Glu-42, Arg-44, Pro-70, Ser-71, Thr-105, Glu-107, and Glu-110 (Figure 8I) and (S2 Figure 3).

**FIGURE 9:**
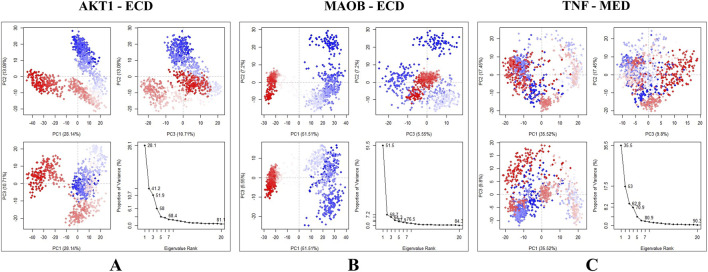
Principal component analysis of the docked complexes, with the blue region indicating the most significant movements, the white region showing intermediate movements, and the red region representing the least flexibility. **(A)** AKT1 - 24-ethylcholesta-5,22-dienol complex shows the variation explained by PC1 (28.14%), PC2(13.08%), and PC3(10.71%). **(B)** Complex MAOB-24-ethylcholesta-5,22-dienol demonstrating the first three principal components (PC1, PC2, PC3) and the scree plot. **(C)** TNF-4alpha-methyl-24-ethylcholesta-7,24-dienol complex, results indicate that the majority of conformational changes occurred in the first three principal components.

Trajectory analysis using PCA revealed conformational changes across all clusters in each of the three docked systems. In the AKT1 - 24-ethylcholesta-5,22-dienol complex, the overall motion was more distributed across several modes with PC1, PC2, and PC3 accounting for 28.14%, 13.08%, and 10.71% of the total motion, respectively. Additionally, the sharp drop in the scree plot after PC5 suggests that the majority of meaningful conformational changes are captured by the first five principal components (Figure 9A). The MAOB-24-ethylcholesta-5,22-dienol complex exhibited the top five eigenvectors in the system, exhibiting notable movements, with eigenvalues ranging from 5.55% to 51.51%. The variances explained by PC1, PC2, and PC3 were 51.51%, 7.2%, and 5.55%, respectively (Figure 9B). The PC1 value reflects the significant conformational change that occurs early in the simulation, as indicated by the RMSD plot. PC2 and PC3 describe additional conformational changes, with PC2 and PC3 describing less dominant, potentially localized motions. These results suggest that the compound binding did not significantly alter the flexibility of the protein residues. For the TNF-4alpha-methyl-24-ethylcholesta-7,24-dienol complex, the first three principal components collectively accounted for 62.77% of the total motion. PC1 explained 35.52% of the variance, indicating a significant conformational shift during the initial nanoseconds of the simulation. PC2 contributes 17.45%, suggesting additional but less pronounced motion, reflecting the onset of structural stability. PC3 (9.8%) reflected minor fluctuations, further supporting the stabilization of the complex (Figure 9C).

The residue cross-correlation matrix was plotted to reveal the correlation between each residue and all the other throughout the simulation period. The positive and negative correlation among residues was analyzed. The AKT1 - 24-ethylcholesta-5,22-dienol complex showed some dark cyan regions in the matrix, indicating that specific residues influenced the movement of other residues. In addition, several white areas stated the presence of independent residues. However, the majority of the matrix remained cyan, indicating minimal correlation, with most residue movements occurring independently (S2 Figure 4A). In the MAOB-24-ethylcholesta-5,22-dienol complex, the majority of the matrix showed a cyan region, suggesting that most residues moved independently without influencing one another. Some areas showed light cyan and white coloring, indicating minor negative correlations. All other residues within the binding pocket demonstrated negligible influence on the movement of neighboring residues during the simulation (S2 Figure 4B). In the TNF-4alpha-methyl-24-ethylcholesta-7,24-dienol complex, several dark cyan regions were observed, indicating notable residue-residue influence. Compared to other complexes, the TNF protein showed more positively correlated regions, suggesting significant coordinated movement across different areas of the protein (S2 Figure 4C).

### 3.7 ADMET analysis

To assess the potential of the compounds identified in this study, their pharmacokinetic properties are analyzed using ADMET Lab 3.0 and TSMSP. Lipinski’s ‘Rule of Five’ (RO5) is used to assess the oral bioavailability and drug-likeness of compounds. LF compounds were initially screened based on MW, LogP, and DL parameters, and those with at least two violations were excluded. It has been observed that 24-methylenecycloartanol ferulate violated MW (617 g/mol) and LogP (10.09 log mol/L) but has a drug-likeness score exceeding 0.30. The final five selected compounds were further assessed for their comprehensive ADMET analyses as mentioned in Table 1.

**TABLE 1:** The ADMET properties of the top 5 compounds.

Compound properties	Cycloartenol	24 -methylene -cycloartenol	Lupeol acetate	24-ethylcholesta-5,22-dienol	4alpha-methyl-24-ethylcholesta-7,24-dienol
Molecular weight (g/mol)	426.8	440.83	468.84	412.77	426.8
log P (log mol/L)	5.722	5.426	7.21	6.737	8.114
Log S (log mol/L)	−5.52	−5.214	−8.773	−6.186	−8.076
H. bond donors	1	1	0	1	1
H. bond acceptors	1	1	2	1	1
OB (%)	38.69	10.4	9.1	43.83	42.3
CaCO_2_ permeability (log cm/s)	−4.908	−5.153	−5.07	−5.317	−4.87
Blood-brain barrier	0.5074	0.3549	0.4591	0.5497	0.3457
Drug likeness	0.78	0.79	0.79	0.76	0.78
Drug-induced neurotoxicity	0.03	0.026	0.149	0.117	0.088
AMES toxicity	0.202	0.226	0.397	0.06	0.144
CYP2D6 inhibitor	0.5–0.7	0.1–0.3	0.0–0.1	0.0–0.1	0.1–0.3
Rotatable bond count	4	5	3	5	5
Fractional absorption surface area	0	0.25	0.24	0.22	0.23
Topological polar surface area (Å^2^)	20.23	20.23	26.3	20.23	20.23
RO5 violations	1	1	1	1	1

**FIGURE 10:**
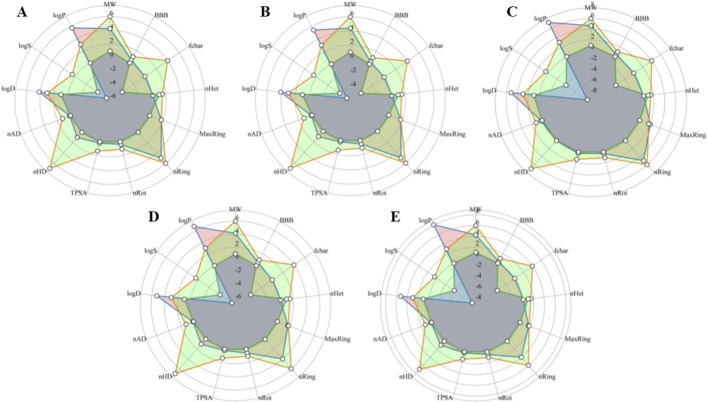
Radar graph shows the ADMET properties of the top five scrutinized compounds. The orange color represents the upper limit of each property, the green color represents the lower limit, and the blue color represents the compound properties. **(A)** Cycloartenol. **(B)** 24-Methylenecycloartanol. **(C)** Lupeol acetate. **(D)** 24-ethylcholesta-5,22-dienol. **(E)** 4alpha-methyl-24-ethylcholesta-7,24-dienol.

Lipinski’s RO5 indicates that all final compounds meet the criteria, except for log P. These compounds have a good chance of crossing the blood-brain barrier (BBB), which is crucial for drugs targeting brain-associated diseases such as PD, given that the brain is considered a fat-rich tissue. In addition, most compounds exhibit low drug-induced neurotoxicity, suggesting they are less likely to affect the nervous system. Low values of the Topological polar surface area also suggest that selected compounds can cross the BBB easily. Collectively, these results indicate that these compounds have potential for use in PD drug development. The ADMET properties of the top 5 compounds are also depicted in the radar graph (Figure 10).

## 4 Discussion

PD is a prevalent and complex neurodegenerative disorder that primarily impacts older adults. Although the exact causes of PD development remain unclear, several factors are associated with the pathology, such as protein aggregation, neuroinflammation, oxidative stress leading to mitochondrial dysfunction, and mutations in specific genes, which are thought to contribute to PD (Lin et al., 2021). Researchers aim to develop potent therapies that will cure or slow down PD progression (Tan et al., 2022). Recent research focused on the pharmacological composition of LF and its bioactive compounds, such as phenolics, polysaccharides, and carotenoids, which were found to exhibit significant antioxidant potential (Neelam et al., 2021; Wang et al., 2023).

Since earlier studies have identified LF as an herb potentially helpful in treating PD, this research aims to uncover its molecular mechanisms against PD through a network pharmacology approach, with molecular docking used to verify the findings. LF fruits are widely utilized owing to their beneficial polysaccharides and phytochemicals, including carotenoids and phenolic compounds, which demonstrate health-promoting effects such as anti-inflammatory and antioxidant properties (Vidović et al., 2022). Many pharmacological studies have found that LF possesses anti-neurodegenerative, anti-aging, and neuroprotective properties (Zhang and Wang, 2025). The most commonly studied group is water-soluble glycoconjugates known as L. barbarum polysaccharides (LBP) (Amagase and Farnsworth, 2011). Carotenoids are another significant group of LF components, especially zeaxanthin. Additionally, LF can serve as a source of phenolic compounds, including phenolic acids and flavonoids. These include quercetin-diglucoside, kaempferol-3-O-rutinoside, caffeic acid, and rutin, which exhibit potent antioxidant activity (Ma et al., 2019). However, the crude extract and the large molecule of LBP raise their bioavailability as an issue for use *in vivo* studies (Amagase and Farnsworth, 2011). A study of pharmacokinetics and excretion of Lycium barbarum polysaccharides in Rats found that LBP might mainly act on the intestinal tract due to its difficulty in being absorbed (Xia et al., 2021). The present study focused on small-molecule phytochemicals with well-defined chemical structures, which are generally more amenable to pharmacokinetic and molecular docking analyses. Phytochemicals with lower molecular weights exhibit higher oral bioavailability than LBPs.

A total of 52 targets were identified as PD-related and interacted with 66 active LF compounds. The potential targets were chosen as AKT1, MAOB, TNF, IL6, and IL1B based on node degree values in the PPI network and the literature.

AKT1 has been linked to dopamine-related neurological diseases because it influences dopamine-related behaviors and participates in the dopamine signaling pathway (Du et al., 2020). Hang et al. reported that AKT plays a crucial role in multiple signaling pathways. These targets include transcription factors, proapoptotic proteins, and protein kinases (Hang et al., 2022). Numerous studies have demonstrated that activation of the cell survival pathway PI3K-AKT may enhance neuroprotection and inhibit oxidative stress, inflammation, and apoptosis induced by mitochondrial disruption (Shen et al., 2022). MAOB, a monoamine oxidase, contributes to the development of neurodegeneration and neuroinflammation in PD and AD. Reactive astrocytes in the substantia nigra exhibit an elevated release of MAOB in PD patients (Nam et al., 2022). Reversible and irreversible MAOB inhibitors have been prescribed for PD patients who suffer from motor symptoms (Tsuboi et al., 2022). Studies have shown that MAOB inhibitors can reduce alpha-synuclein aggregation by blocking the formation of sheet-like and linear alpha-synuclein structures required for aggregation (Sekar et al., 2024). MAOB plays a key role in reducing dopamine levels by breaking it down, thereby elevating oxidative stress and leading to the degeneration of dopaminergic neurons. It is also crucial in the synthesis of γ-aminobutyric acid (GABA), which inhibits adjacent dopaminergic neurons, thereby reducing dopamine levels in the brain and contributing to the progression of PD (Singh et al., 2024).

TNF is a pro-inflammatory cytokine produced by microglial cells in response to brain injury, infections, and neurodegenerative diseases such as PD and AD (Amin et al., 2022). Studies indicate that TNF induces neuroinflammation and the degeneration of dopaminergic neurons through programmed cell death. Furthermore, the continual synthesis of alpha-synuclein will stimulate the release of various neurotoxic and pro-inflammatory factors, including TNF, which adversely impact neurons (Zhao and Yang, 2021). Although neuroinflammation serves as a neuroprotective mechanism, prolonged neuroinflammatory responses are associated with neurodegeneration and may induce neurotoxicity, with TNF being implicated in both processes (Kwon and Koh, 2020). IL6 is another pro-inflammatory cytokine present in the brain, typically at low levels; however, its synthesis increases substantially during PD and other neurological disorders. IL6 can promote cell survival by acting as a neurotrophic factor, but it can also cause neuronal degeneration (Kummer et al., 2021). A meta-analysis focusing on PD inflammatory markers has identified a significant increase in IL6 production among patients with PD (Qu et al., 2023). Other studies have also identified elevated levels of IL6 in the postmortem brains of PD patients, particularly within the substantia nigra region (Pons-Espinal et al., 2024). IL1B, a pro-inflammatory cytokine, was found to be elevated in dopaminergic tissues of PD patients. Additionally, high expression of IL1B caused by specific mutations seems to be linked to a higher risk of developing PD (Pike et al., 2021). High expression of IL1B within the central nervous system (CNS) is believed to induce neuronal damage, which is associated with numerous neurological disorders, such as PD (Mendiola and Cardona, 2018).

GO and KEGG enrichment analyses were conducted to identify the biological processes (BP), molecular functions (MF), cellular components (CC), and pathways of the 52 common targets to elucidate their roles in neurodegenerative disorders, particularly PD. BP analysis shows that synaptic signaling is among the most significant terms, and the effect of α-synuclein on synaptic vesicle recycling is profoundly altered at the onset of PD. Experiments have demonstrated that synaptic dysfunction could arise from postsynaptic modifications in PD patients’ striatum driven by excessive dopaminergic loss (Picconi et al., 2012). MF analysis of common targets highlights primary associations with neurotransmitter receptor activity, oxidoreductase activity, dopamine binding, and postsynaptic neurotransmitter receptor activity pathways, all of which are significantly related to PD. The CC terms, including neuron projection, neuronal cell body, synapse, and mitochondria, were among the top 10 enriched terms associated with targeted genes. Previous genetic investigations highlighted mitochondria’s key role in the progression of neurological deterioration in PD, since several PD-related genes code for mitochondrial homeostasis proteins (Grünewald et al., 2019).

KEGG enrichment analysis identified the AD, dopaminergic synapse, and neurodegeneration-multiple diseases pathways as significant, with common recruited targets, suggesting potential roles for these genes in the mechanism of neurodegenerative disease progression. Although AD and PD affect different brain regions and exhibit various neurological features, studies show they share many similarities in the progression of neuronal decline, including common factors such as genes, alpha-synuclein, oxidative stress, and mitochondrial dysfunction (Monzio Compagnoni et al., 2020; Boyd et al., 2022). CASP3, TNF, and IL6 are involved in processes such as apoptosis, oxidative stress, ROS formation, axonal transport defects, and age-related signaling pathways associated with diabetic complications in multiple neurodegenerative diseases. The dopaminergic synapse is another significant pathway highlighted by KEGG analysis. MAO is directly involved in the degradation of dopamine in neurons (presynaptic terminal), and AKT inhibits GSK-3 in synaptic plasticity. Previous research has shown that dopamine neuron loss associated with PD begins in synaptic terminals and axons and then extends to the substantia nigra (Morales et al., 2015).

To validate the study’s findings, molecular docking was conducted, revealing that the proteins AKT1, TNF, and MAOB exhibit high binding affinity for LF compounds. The lupeol acetate exhibits the least binding energy with all top target proteins, followed by cycloartenol and 24-ethylcholesta-5,22-dienol. Lupeol acetate, a derivative of lupeol, a pentacyclic triterpenoid, shows potent anti-inflammatory and antioxidant properties and notable BBB permeability (Park et al., 2023). Lupeol acetate helps in regulating proteins involved in synaptic dysfunction, indicating its potential for treating PD (Choe et al., 2024).

MD simulation is a widely employed computational method for characterizing biological systems. It enables the investigation of lipid–protein interactions, membrane structure and organization, protein conformational dynamics, and protein–ligand interactions (Filipe and Loura, 2022). To assess the equilibration and structural stability of a protein–ligand complex, RMSD is calculated, providing insights into the protein’s conformational changes throughout the simulation. Additionally, RMSF is used to evaluate the flexibility of individual residues, where higher peaks in the RMSF plot indicate more flexible regions of the protein. Hydrogen bond analysis is also performed, as the presence of hydrogen bonds contributes significantly to the stability of the protein–ligand complex (Ghahremanian et al., 2022).

The top compounds with good binding to all five targets were analyzed and selected for further MD simulations to assess stability and structural fluctuations. MD simulation analyses of the current study. The results show that the AKT1 - 24-ethylcholesta-5,22-dienol complex exhibits good stability, with only minor fluctuations observed in the RMSD and RMSF plots. Approximately six hydrogen bonds were identified in the protein-ligand interactions, which remained stable throughout the simulation. The MAOB-24-ethylcholesta-5,22-dienol complex exhibited minor fluctuations at the beginning of the simulations, likely due to conformational adjustments as the protein and ligand approached equilibrium. Three hydrogen bonds were observed, along with ionic and hydrophobic interactions. In contrast, the TNF-4alpha-methyl-24-ethylcholesta-7,24-dienol complex exhibited ligand fluctuations of up to 14 Å, indicating poor stability. Furthermore, most interactions within this complex persisted for <30% of the simulation duration. These fluctuations may result from ligand binding to the protein’s outer surface.

PCA showed that the first three principal components accounted for the majority of the total motion of the complexes, reflecting the most pronounced conformational changes during the simulation’s initial phase. The distribution of variance among the top principal components reflects stable protein conformations while also showing the flexibility necessary for biological function. The residue cross-correlation matrix revealed that the TNF-4alpha-methyl-24-ethylcholesta-7,24-dienol complex contained the highest number of positively correlated residues, indicating coordinated movements between these regions. In contrast, the other complexes showed only minor positive and negative correlations, suggesting that most residues moved independently of one another.

ADMET and physicochemical properties were predicted and analyzed to investigate the suitability of selected compounds for drug development. Initially, ADMET screening identified only one compound with both MW and logP values exceeding thresholds but with good drug likeness. The ADMET analysis of 66 compounds, identified through target interactions, was also conducted. Overall, these compounds exhibit favorable ADMET properties, except for rutin, which shows variation in MW, HBD, and HBA.

Lipinski’s RO5 states that a compound should have a molecular weight less than 500, less than 5 HBD, 10 HBD, and a log P value less than 5 for better oral absorption and overall drug likeness (Lipinski, 2001). All the top 5 compounds have met the criteria, although the log P exceeded the limit of 5. BBB permeability is significant in PD, as therapeutic agents must cross this barrier to influence neuronal pathways involved in disease progression. The compounds show moderate BBB permeability and relatively low TPSA values, suggesting that the selected phytochemicals may have a reasonable likelihood of reaching brain tissue. Lupeol acetate exhibits good drug likeness (0.79), moderate BBB permeability (0.4591), and low neurotoxicity risk (0.149). Cycloartenol also shows potential as a therapeutic agent for PD, with a favorable drug-likeness score of 0.78, a minimal drug-induced neurotoxicity level of 0.03, and an AMES toxicity rating of 0.202. Cycloartenol is a phytosterol molecule involved in various sterol syntheses with a range of pharmacological properties, including anti-tumor, antibiosis, anti-inflammatory, antioxidant, and anti-AD properties (Zhang et al., 2017). Although research on cycloartenol’s effects on PD remains limited, its properties underscore its potential as a therapeutic agent for PD.

A current study focused on identifying potential PD targets using a network pharmacology approach, followed by the identification of promising LF-derived compounds. Extensive computational methods, including PPI networks, docking, ADMET, and simulation analyses, have been conducted to identify drug-like compounds for effective therapies against PD. Although computational approaches provide a strong basis for identifying potential therapeutic compounds, *in vitro* and *in vivo* validation is still required to verify their safety and effectiveness in a physiological setting. Future research should focus on improving the pharmacokinetics and bioavailability of these drugs through structural optimization or advanced drug-delivery methods. Furthermore, investigating how these substances interact with current treatments may enhance their therapeutic efficacy. Current findings are crucial, followed by experimental validation to translate them into tangible therapeutics for PD.

## 5 Conclusion

The study uncovers how *Lycii Fructus* exerts its anti-Parkinson’s disease effects and identifies potential targets and active compounds for therapy. Bioinformatics analyses identified potential hub genes, including AKT1, IL1B, TNF, IL6, and MAOB, using drug-target and PPI networks, followed by GO and KEGG analyses. Molecular docking and ADMET analysis have identified potential active compounds, including lupeol acetate, cycloartenol, 24-ethylcholesta-5,22-dienol, 24-methylene-cycloartenol, and 4alpha-methyl-24-ethylcholesta-7,24-dienol. These compounds exhibited promising binding potential for the scrutinized hub gene, particularly AKT1. It has been concluded that AKT1, IL1B, TNF, IL6, and MAOB have good potential for targeting these LF-active compounds, which could be used to develop novel, effective drugs for PD. The pharmacokinetic properties also suggest that these compounds encompass drug-likeness potential and are promising novel candidates for effective drug development against PD. *In vitro* experiments and biological assays are required to validate the biological activity of these compounds and support animal trials in drug discovery and development.

## Data Availability

The original contributions presented in the study are included in the article/Supplementary Material, further inquiries can be directed to the corresponding author.
