# Supplementary material for: Network pharmacology-based identification of potential drug targets and bioactive compounds in Lycii Fructus (Gouqizi) for the therapeutics of Parkinson’s disease
Source: Front Pharmacol. 2026 Jan 12;16:1714071. doi: 10.3389/fphar.2025.1714071 (PMC12833033; doi:10.3389/fphar.2025.1714071)
Supplement: Supplementary file 1 [file DataSheet2.docx]

Supplementary Material S2

**Network Pharmacology-Based Identification of Potential Drug Targets and Bioactive Compounds in *Lycii Fructus* (Gouqizi) for Parkinson’s Disease Treatment**

Supplementary Figure 1. The interacting residues throughout the simulation with the ligand at each trajectory frame of the complex AKT1-24-ethylcholesta-5,22-dienol including H-bonds, hydrophobic, ionic, and water bridges contacts.

Supplementary Figure 2. A timeline representation of the interactions and contacts (H-bonds, Hydrophobic, Ionic, Water bridges) between protein residues and ligand in each trajectory frame of the complex MAOB-24-ethylcholesta-5,22-dienol.

Supplementary Figure 3. The interacting residues throughout the simulation of the complex TNF-4alpha-methyl-24-ethylcholesta-7,24-dienol at each trajectory frame. These contacts include H-bonds, hydrophobic, ionic, and water bridges interactions.


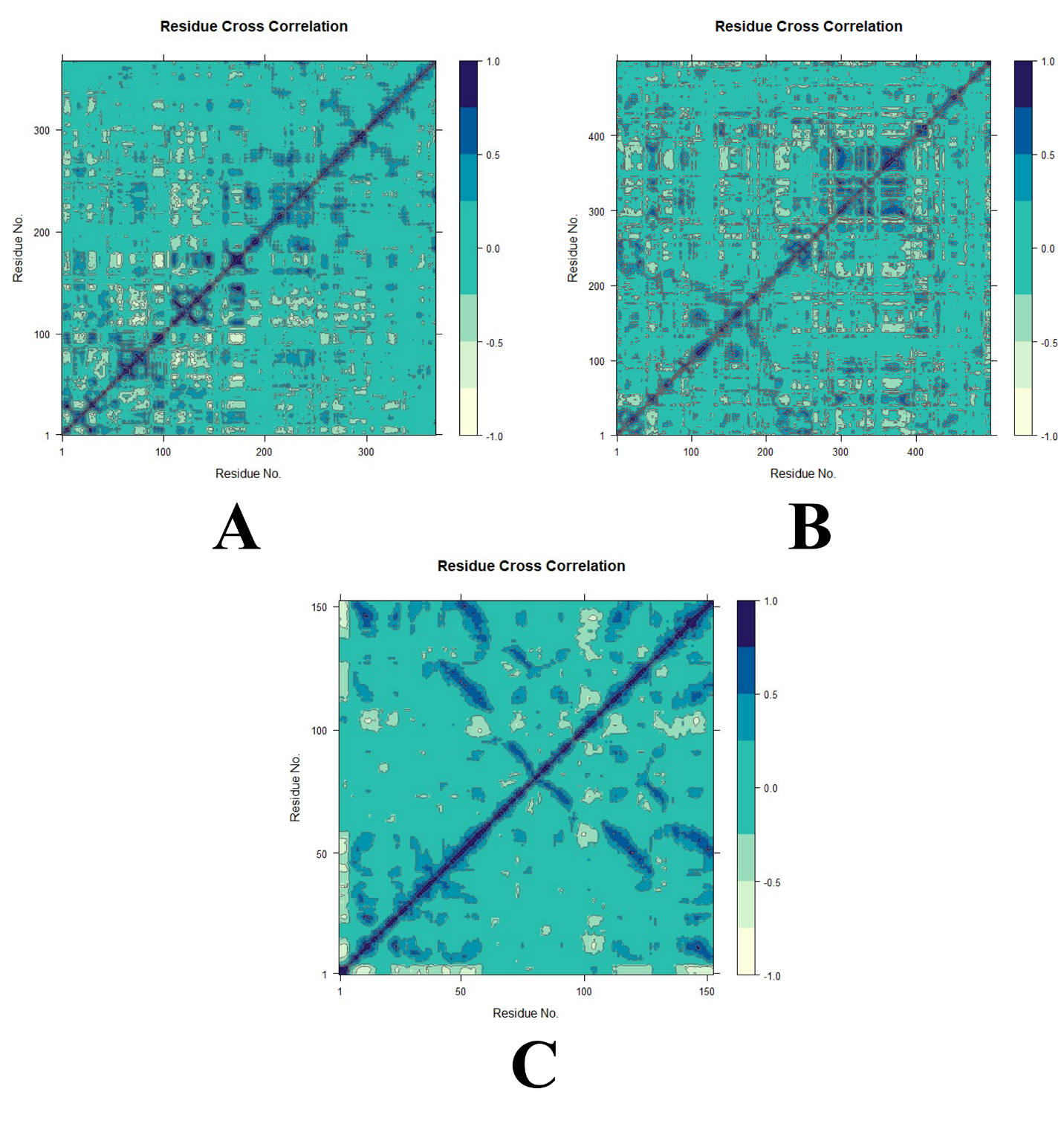


Supplementary Figure 4. Residue cross correlation analysis results. A) AKT1 - 24-ethylcholesta-5,22-dienol. B) MAOB - 24-ethylcholesta-5,22-dienol. C) TNF-4alpha-methyl-24-ethylcholesta-7,24-dienol. The X-axis shows the residues, while RMSF (Å) is presented on the y-axis. Dark cyan regions indicated positive correlation between residues. Cyan color (0.0) indicated there is no correlation between residues, while white regions indicate negative correlation between residues.q
