# Supplementary material for: Network pharmacology-based identification of potential drug targets and bioactive compounds in Lycii Fructus (Gouqizi) for the therapeutics of Parkinson’s disease
Source: Front Pharmacol. 2026 Jan 12;16:1714071. doi: 10.3389/fphar.2025.1714071 (PMC12833033; doi:10.3389/fphar.2025.1714071)
Supplement: Supplementary file 2 [file DataSheet1.docx]

**Supplementary Material S1**


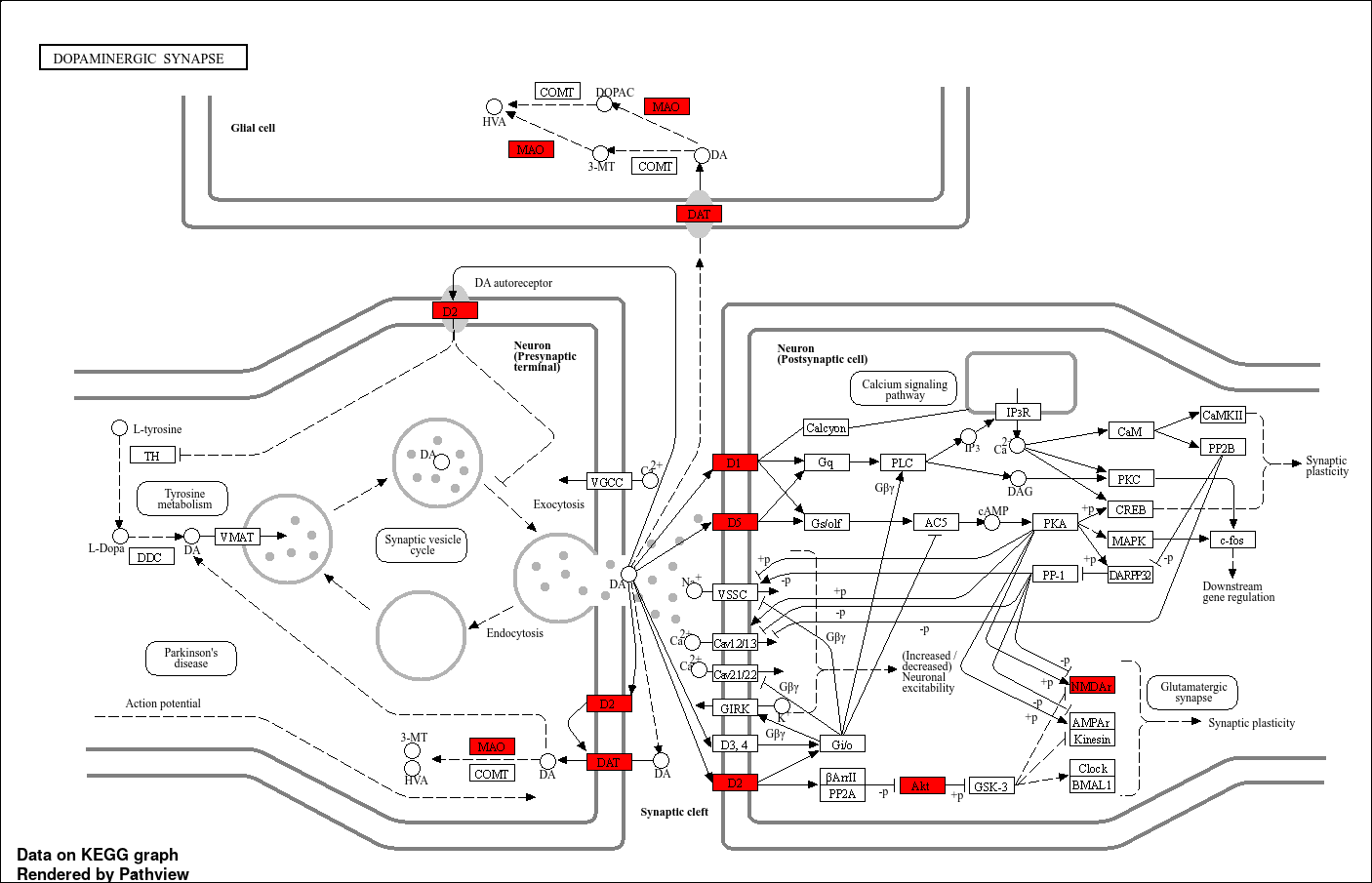
**Network Pharmacology-Based Identification of Potential Drug Targets and Bioactive Compounds in *Lycii Fructus* (Gouqizi) for Parkinson’s Disease Treatment**

**Figure 1:** Dopaminergic synapse pathway, potential targets of PD are highlighted in red.


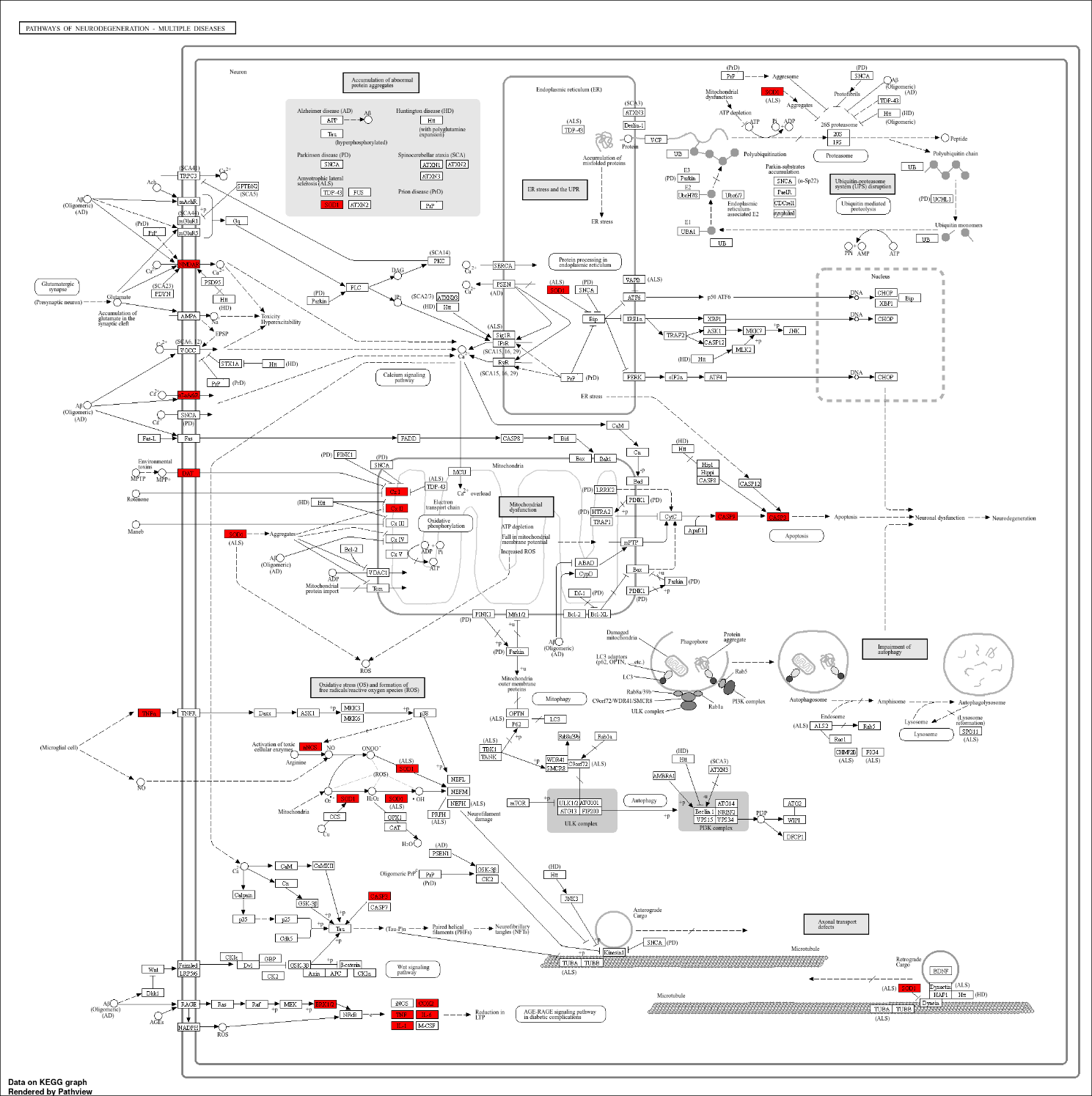


**Figure 2:** Pathways of neurodegeneration-multiple diseases, red genes represent potential targets of PD associated with these pathways.


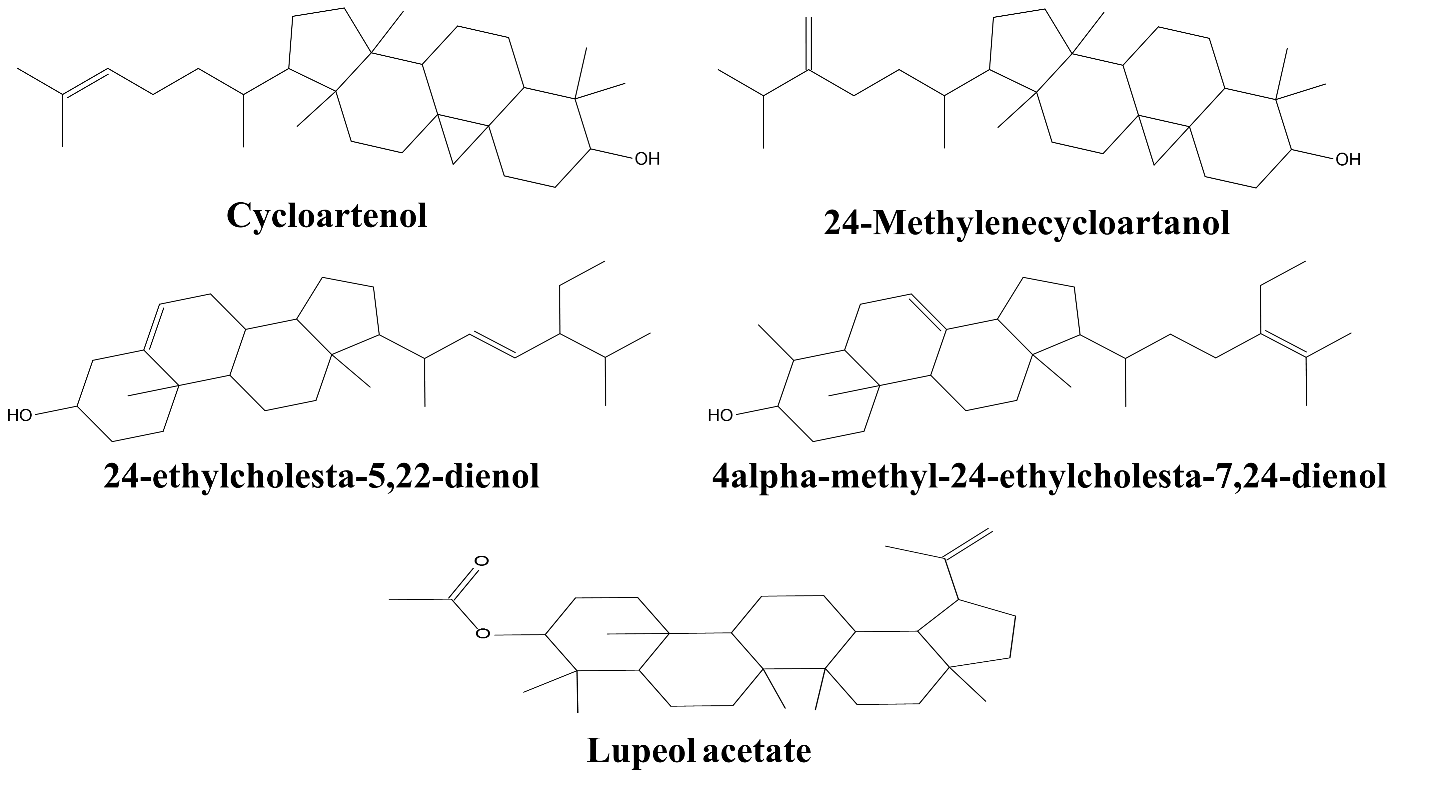


**Figure 3:** 2D chemical structure of the top 5 compounds of Lycii fructus.


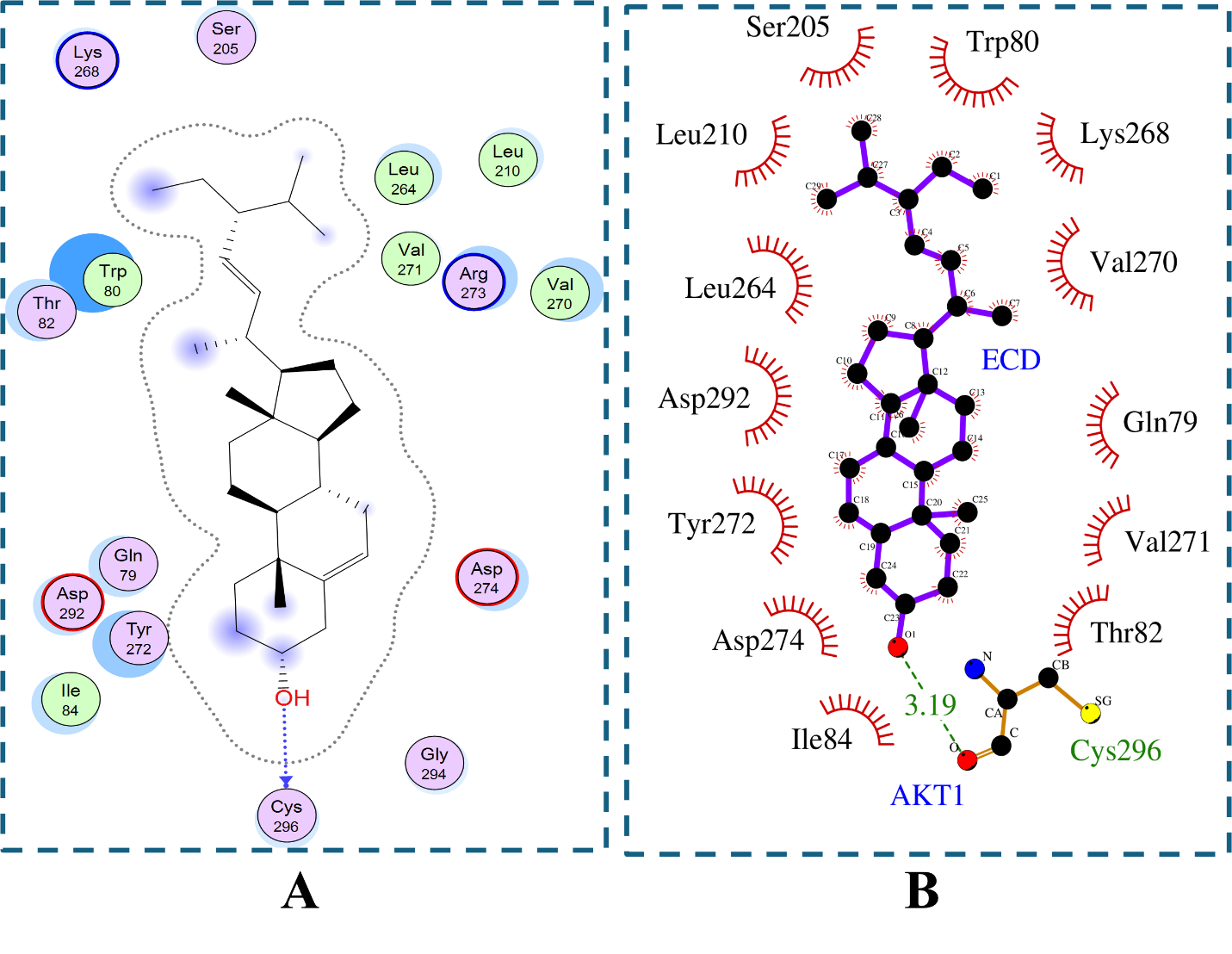


**Figure 4:** Interacting residues of the complex AKT1-24-ethylcholesta-5,22-dienol (ECD). **A)** Type of interacting residues generated by MOE; light purple indicates polar residues, and green indicates non-polar residues. Polar residues with a blue border refer to basic residues, while red border refers to acidic residues. **B)** Interaction analysis by LigPlot+; green labels represent hydrogen bonds, while red residues represent hydrophobic interactions.


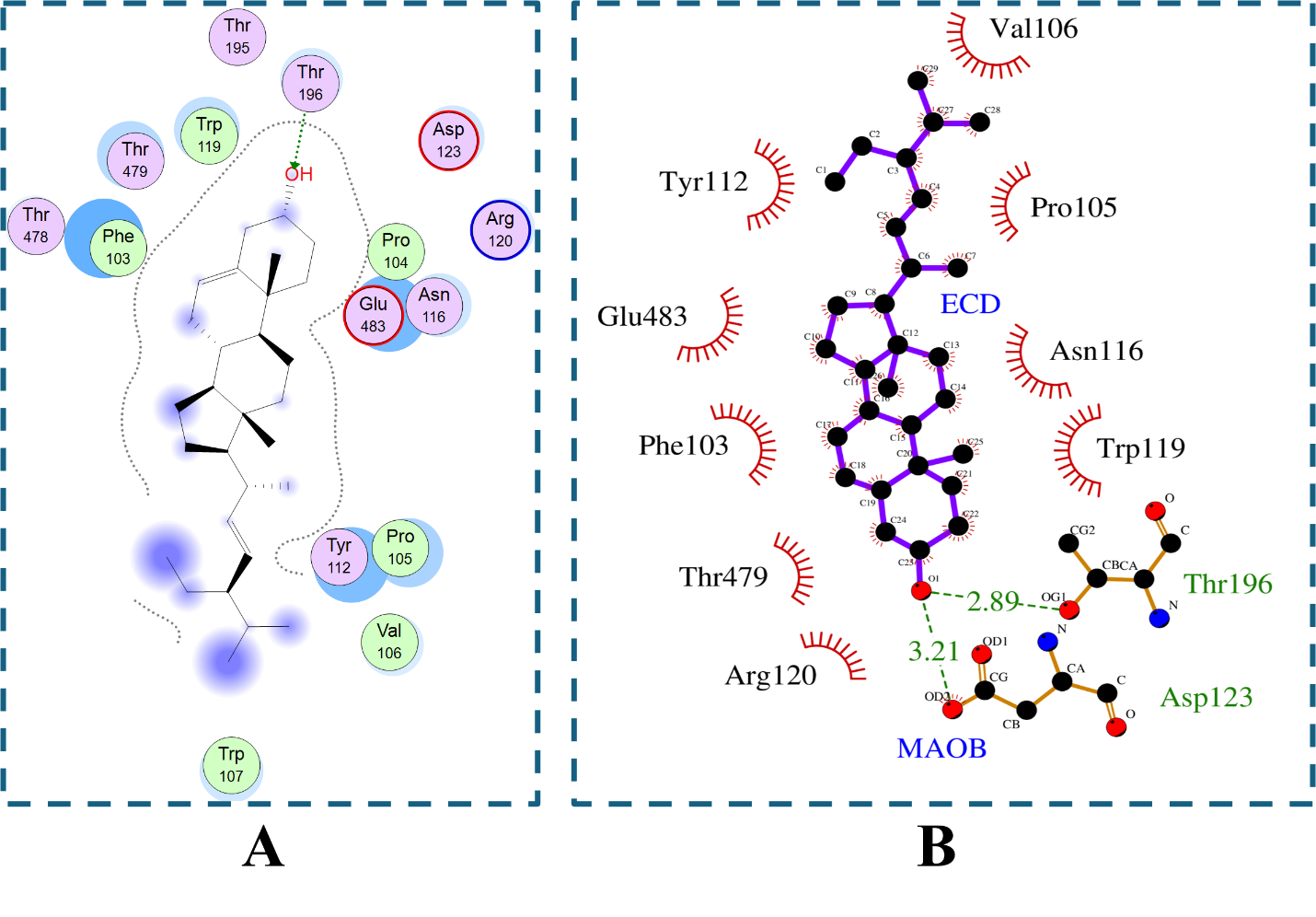


**Figure 5:** Residue interactions analysis of MAOB-24-ethylcholesta-5,22-dienol (ECD) complex. **A)** Interacting residues classification by MOE. Light purple represents polar residues, while green indicates non-polar residues. Polar residue with a red border refers to acidic residue, while a blue border refers to basic residue. **B)** Interacting residues analysis by LigPlot+; green labels represent hydrogen bonds along with the distance, while red residues represent hydrophobic interactions.


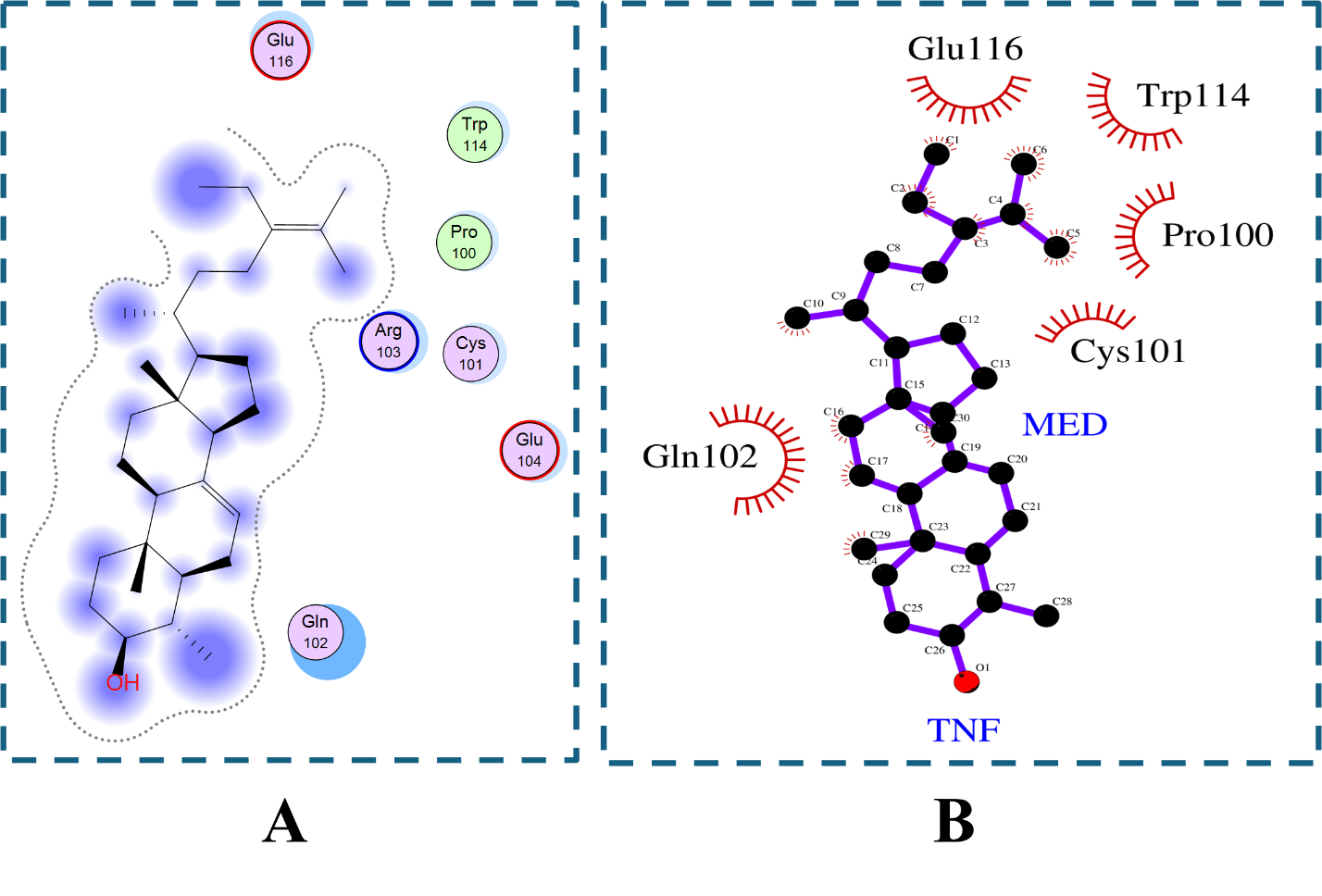


**Figure 6:** Interacting residues of the complex TNF-4alpha-methyl-24-ethylcholesta-7,24-dienol (MED). **A)** Type of interacting residues generated by MOE; green indicates non-polar residues while light purple indicates polar residues. Polar residues with a blue border refer to basic residues, while red border refers to acidic residues. **B)** Interaction analysis by LigPlot+; red residues represent hydrophobic interactions.
